# Assuring access to topical mosquito repellents within an intensive distribution scheme: a case study in a remote province of Cambodia

**DOI:** 10.1186/s12936-015-0960-4

**Published:** 2015-11-24

**Authors:** Somony Heng, Lies Durnez, Charlotte Gryseels, Karel Van Roey, Vanna Mean, Sambunny Uk, Sovannaroth Siv, Koen Peeters Grietens, Tho Sochantha, Marc Coosemans, Vincent Sluydts

**Affiliations:** National Center for Parasitology, Entomology and Malaria Control, Phnom Penh, Cambodia; Institute of Tropical Medicine, Antwerp, Belgium; University of Antwerp, Antwerp, Belgium; School of International Health Development, Nagasaki University, Nagasaki, Japan; Partners for Applied Social Sciences (PASS) International, Tessenderlo, Belgium

**Keywords:** Distribution, Access, Malaria, Repellents

## Abstract

**Background:**

The public health value of a vector control tool depends on its epidemiological efficacy, but also on its ease of implementation. This study describes an intensive distribution scheme of a topical repellent implemented in 2012 and 2013 for the purpose of a cluster-randomized trial using the existing public health system. The trial aimed to assess the effectiveness of repellents in addition to long-lasting insecticidal nets (LLIN) and occurred in a province of Cambodia. Determinants for accessibility and consumption of this tool were explored.

**Methods:**

135 individuals were appointed to be repellent distributors in 57 villages. A 2-weekly bottle exchange programme was organized. Distributors recorded information regarding the amount of bottles exchanged, repellent leftover, and reasons for not complying in household data sheets. Distributor-household contact rates and average 2-weekly consumption of repellent were calculated. Household and distributors characteristics were obtained using questionnaires, surveying 50 households per cluster and all distributors. Regression models were used to explore associations between contact and consumption rates and determinants such as socio-economic status. Operational costs for repellent and net distribution were obtained from the MalaResT project and the provincial health department.

**Results:**

A fourfold increase in distributor-household contact rates was observed in 2013 compared to 2012 (median_2012_ = 20 %, median_2013_ = 88.9 %). Consumption rate tripled over the 2-year study period (median_2012_ = 20 %, median_2013_ = 57.89 %). Contact rates were found to associate with district, commune and knowing the distributor, while consumption was associated with district and household head occupation. The annual operational cost per capita for repellent distribution was 31 times more expensive than LLIN distribution (USD 4.33 versus USD 0.14).

**Discussion:**

After the existing public health system was reinforced with programmatic and logistic support, an intense 2-weekly distribution scheme of a vector control tool over a 2-year period was operated successfully in the field. Lack of associations with socio-economic status suggested that the free distribution strategy resulted in equitable access to repellents. The operational costs for the repellent distribution and exchange programme were much higher than LLIN distribution. Such effort could only be justified in the context of malaria elimination where these interventions are expected to be limited in time.

**Electronic supplementary material:**

The online version of this article (doi:10.1186/s12936-015-0960-4) contains supplementary material, which is available to authorized users.

## Background

During the past decade an increase in the use of long-lasting insecticidal nets (LLINs) and indoor residual spraying (IRS) has contributed to an unprecedented decrease in the worldwide malaria burden [1–3]. However, the presence of early and outdoor biting malaria vectors, responsible for residual malaria transmission [4–7] hamper further reductions in malaria transmission in many endemic areas. In a low transmission setting, a subsequent reduction in malaria transmission will require public health programmes to address this residual transmission [8]. Several additional vector control tools have been suggested for targeting residual transmission, such as the use of topical and spatial repellents [9], insecticide-treated veils or wraps [10], clothes [11] and long-lasting insecticidal hammocks [12]. These additional vector control tools are mostly based on personal protection, and were targeted for use by at-risk populations experiencing higher exposure to malaria-infected mosquitoes [9]. Region-wide application of such tools in public health programmes may require more intensive distribution schemes as compared to LLINs and IRS.

The public health value of a vector control tool does not only depend on its epidemiological efficacy, but also on its ease of implementation as well as its acceptability and use by the human population, all of which translate into the effective coverage of the tool. For IRS, effective coverage depends on the proportion of houses correctly treated with insecticides while other control tools require the active participation of the target population. IRS has to be repeated every 3–6 months, whereas its use does not require any further active commitment of the target population beyond preparing the house the day of the spray and not washing or plastering the walls after the spray round. On the other hand, LLIN distribution needed to be organized at a 3-year interval only as the bio-efficacy of those nets last at least 3 years. However, for an LLIN to be effective, the target population has to sleep under the nets, and therefore its effective coverage will strongly depend on its effective daily use [13]. In many countries, mainly on the African continent, different approaches have been implemented in public health systems to reach sufficient accessibility and daily use of LLINs, both defining coverage. For example, continuous distribution of LLINs was integrated in other public health interventions such as measles and polio vaccination in Mozambique, Zambia and Ghana [14–16]. In addition, several ways to increase the uptake of malaria preventive measures through community involvement are used such as hang-up keep-up strategies [17], health promotion [18], and participatory approaches relying on individual [19] and community involvement. In order to better understand how access to, and use of, preventive measures to control malaria could be improved, several studies have looked into determinants of ownership such as socio-economic status (SES), demography and geography for the distribution and use of LLINs [20–26].

Many of the suggested additional vector control tools targeting residual transmission were initially designed for personal protection against mosquito nuisance and thus require repeated individual use. A different public health approach is required for large-scale implementation of these tools and needs for example including intensive distribution schemes or more profound health education systems. In many countries, networks of village health workers [27], such as the Village Malaria Workers (VMWs) [28, 29] and Village Health Support Group (VHSG) in Cambodia have been set up to assist in public health programmes. Integration of intensive distribution and health education systems in these networks might be feasible and cost-effective in the context of a malaria elimination strategy where sustainability is less a concern and where such networks were already in place.

The Royal Government of Cambodia is very much engaged in achieving malaria elimination by 2025 [30]. The Cambodian health system, which relies on VMWs and VHSGs, is particularly well-suited to serve as backbone to the integration of an extensive distribution programme. The National Malaria Control Programme of Cambodia supports the network of VMWs in all villages with a relatively high malaria incidence to provide diagnosis and treatment at the village level. These VMWs are also actively involved in the distribution of LLINs and the spread of health information in the villages. The installation of this VMW network has contributed to reducing the malaria burden [31]. VHSGs, created by ministry of health, provide a link between community and health facilities [32].

In this study, which is part of a cluster randomized trial to evaluate the epidemiological efficacy of topical repellents in addition to LLINs at the community level (MalaResT; registered as NCT01663831), the effectiveness of an extensive distribution scheme of topical repellents integrated into the VMW and VHSG systems was evaluated. The study established the feasibility of implementing intensive (with contact every 2 weeks) distribution schemes of additional vector control tools, in this case topical repellents, into the existing public health systems but with staff reinforcement. The impact of increased distribution and health education campaigns on the repellent consumption as well as the influence of different determinants to the distribution of the topical repellent were evaluated. The annual operational cost per capita of repellent distribution was calculated and compared with that of LLIN distribution in the same setting, recognizing that LLINs are effective during 3 years. The results presented could be viewed as an intermediate step towards operational implementation of intensive distribution schemes outside trial conditions.

## Methods

### Study area and population

The trial was conducted in 113 villages (grouped into 98 clusters) in Ratanakiri province, the northeast of Cambodia, from January 2012 to December 2013. The study area is mountainous and mostly either covered by forest or deforested [33]. People in the area belong to 10 different minority groups, and are mostly subsistence farmers cultivating seasonal crops such as dry rice, cassava and beans on slash-and-burn forest fields [34]. In most villages, a large number of households have farms outside the villages where they usually work and stay in plot huts during planting or harvesting season. Here they are more exposed to malaria, but preventive materials such as mosquito nets are usually present [8, 34]. Road conditions between the villages and the farms are generally poor especially in the rainy season (June–October) when some villages and farms are only accessible by boat and or on foot.

In both arms of the MalaResT project, LLIN distribution was carried out by the Provincial Malaria Control Programme and supervised by the National Malaria Centre (CNM), aiming to cover 100 % of the population with a ratio of one net to one person regardless of age. In the intervention arm, all participants aged from 2 to 10 years were additionally provided with 10 % Picaridin topical repellent (lotion), and over 10 years old with 20 % Picaridin (spray). No placebo repellent was provided in the control arm.

### Strategy for repellent distribution and promotion of repellent use

**Fig. 1:**
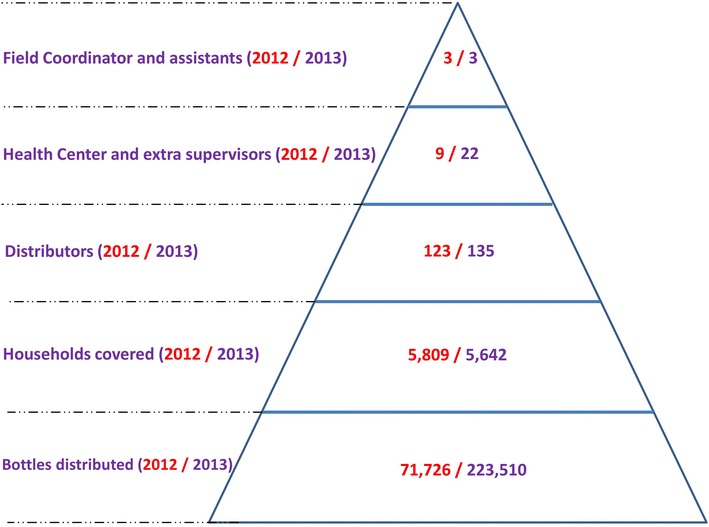
Pyramidal system for repellent distribution and supervision. Number of people involved in the trial by level, number of households covered by the trial and number of repellent bottles distributed in 2012 (*red*) and 2013 (*purple*)

A bottle of repellent contains 100 ml of repellent (spray or lotion), which is the amount required for two applications per day during 2 weeks for an adult (>10 years, spray) and during 4 weeks for a child (2–10 years, lotion). To fit the existing VMW and VHSG systems and the repellent application rates, a pyramidal distribution strategy was opted for with a 2-weekly bottle exchange schedule (Fig. 1). All 57 intervention villages, grouped into 49 clusters, had an individual belonging to the VHSGs and 44 of them had VMWs in place. Many of VMWs and VHSGs were one and the same person. All VMWs and 13 individuals from the VHSGs were recruited as distributors during the trial. To complete the ratio of one distributor for every 50 households in a village, an additional selection of 66 distributors was made in 2012. Motivation and ability to read and write Khmer language were key criteria for selection. Compensations for the distributors’ work existed of per-diems and travel costs during repellent distribution times in the village and travel costs to 2-weekly meetings at the health centre (HC). Each distributor was assigned to work for households near his/her house in the village or on the farm. In 2013, five inactive distributors were replaced and 12 additional distributors were selected in hard-to-reach villages. All distributors were supervised by HC staff with assistance from extra supervisors and project staff. In 2012, the distributors were supervised by nine HC and two project staff. In 2013, one to two extra supervisors per HC (total 13 extra supervisors), according to the study area size of each HC, were recruited to assist the HC staff to support the distributors in improving the repellent distribution, promoting its use and facilitating data collection.

Every 2 weeks, the distributors exchanged bottles in their respective catchment areas by setting central points to meet household representatives who came with the used bottles. Afterwards, distributors went from house to house in the villages and to the plot huts searching those who were absent. Bottles with less than half of the remaining amount of repellent were exchanged for new ones; otherwise they were given back to the users for further use. All required information was recorded in a household datasheet (HDS). A distribution round required 2–3 days and had to be finished just before the 2-weekly meeting at the HC. In each meeting, steered by HC and project staff or supervisors, HDSs were checked for completeness and quality of the collected information. The empty bottles were collected for recycling by a specialized waste management company. Re-using the old bottles was not an option for safety reasons, as the empty bottles contaminated by repellents may be used for other purposes without changing the label. Moreover refilled bottles may lose their label over time. The distributors were given the required number of new repellent bottles for the next distribution session. In each HC, a separate storing space was refilled with repellents a few days prior to each meeting.

All 13 extra supervisors were employed by the project with a monthly salary of USD 180 per person. Monthly incentive for HC staff of USD 40 per person and their per-diem of USD 5 per day and travel costs to do supervisions were also paid by the project. The routine meeting schedule of VMWs is monthly and VHSGs quarterly. Thus once a month and once a quarter the distributors meeting overlapped with VMWs and VHSGs meetings, respectively. It was estimated that, on average, a distributor worked about 1.75 full days a week in the distribution scheme.

To promote repellent and LLIN use, a series of health education campaigns were conducted between April and May 2012. Leaflets and posters explaining when and how to apply repellents were distributed in the intervention arm and the use of LLINs in both arms. Two applications per day of the repellent were recommended: in the evening (between 5 and 7 pm) and after getting up in the morning. During the health education campaign of March–May 2013, a movie about LLIN and repellent use (the last one only in the intervention arm) was projected on big screen followed by knowledge verification and this in addition to the information provided during the campaign of 2012. Furthermore, in 2013, the supervisors regularly performed house-to-house health education for those who did not use repellent and those who used less.

### Data collection

Two sources of information were used in this study:

1. Household data sheets (HDS)
  In both study years, during each bottle exchange, the distributors interviewed the available household representatives based on a standard fill-in form (quantitative survey) and checked all used bottles (Additional file 1). Following information was recorded in the HDSs: codes of used bottles, amount of repellent leftover in each bottle, adverse events experienced by any household member, reasons for not complying or not using the repellent by any household member, codes of new bottles and dates of bottle exchange. Each household received a unique identification code which was used during the entire project. Data were aggregated at the family level.
  Based on the data obtained in the HDSs, two rates were measured: (1) The ***contact****rateper household per year* relates to the contact between a household and its repellent distributor. (2) The ***consumption****rate of repellent per household per 2* *weeks* relates to the amount of repellent used by a single household over 2 weeks. These rates are defined as follows:
  1 $$\varvec{Contact} = \frac{Observed \,Contacts\, per \,family\, per\, year\, (OC)}{Expected \,Contacts \,per\,family\, per \,year\, (EC)} \times 100$$
  where OC is the *actual* number of contacts between a household and its distributor based on HDSs (one HDS represents one contact).
  EC is the total number of contacts a household should have had with the distributor for bottles exchange based on the 2-weekly schedule over the entire distribution season.
  2 $$\varvec{Consumption } = \frac{average\, actual\, consumption \,per\, two\, weeks}{Expected\, consumption\, per\, two\, weeks } \times 100$$
  The average actual consumption per 2 weeks corresponds to the total amount of repellent (ml) a household received, divided by the expected contacts per year.
  The expected repellent consumption per 2 weeks is 100 ml for an adult (>10 years) and 50 ml for a child (2–10 years). The number of adults and children per household were extracted from the census performed in March 2013.
  The information from MalaResT project and Ratanakiri provincial health department financial reports was used to calculate annual operational cost per capita of repellent and LLIN distribution, respectively.
2. Surveys
  *Household survey* 50 households per cluster from the intervention arm were randomly selected based on the most recent census in 2013. For seven clusters with less than 50 households all of them were included. In total, 2377 households were selected and included in the survey between August and December of 2013. The interviews were conducted with adult household representatives using a pre-tested structured questionnaire (Additional file 2) to collect information on: demography, living duration in the village, household size, number of houses, household (HH) characteristics, occupation of household’s head, possession of assets (i.e. transportation, agriculture equipment, animal, entertainment material, light and power source and agriculture land), current residence, possibility of accessing distributor’s house in the rainy season by motorbike and by boat, travel duration to the distributor’s house, and the way the HH usually received the repellents.
  *Distributor survey* All 135 distributors were interviewed between August and September of 2013 using a structured questionnaire which was pre-tested in the field (Additional file 3). The following variables were assessed: demography, living duration in the village, occupation, number of households they were responsible for, location of the households, available transport, possibility of visiting the houses at farms in the rainy season and how they distributed the repellents.

All data from surveys and HDSs were entered by data-entry-clerks in pretested databases in MS Access 2010. A single entry for data from distributor reports and a double entry for the household survey were done.

### Data analysis

Correlation between contact rates and consumption were modelled using a mixed-effects linear regression model with a fixed variance structure assuming the variance increases with increased contact rates, and separately for each study year. Random slopes were included to allow for variability among the villages.

Each household was ranked within an SES category by using the results of a principal component analysis (PCA) based on 18 household’s durable assets and housing characteristics including the number of owned houses, type of main house, type of wall, type of roof, completeness of wall, completeness of roof, house condition, house size, number of families living in a house, main job of household head, owned transportations, owned agricultural equipment, owned animals, owned entertainment equipment, owned power sources, owned farmland, owned rice field and owned cashew nut farm. Five quintiles of SES levels were determined ranging from lowest to highest as Q1, Q2, Q3, Q4 and Q5 representing poorest, second, middle, fourth and richest.

In order to identify potential determinants for contact and consumption, mixed models were fitted with as response variables respectively contact and consumption and as fixed effect explanatory variables the different potential determinants such as: SES, district, commune, user’s household head occupation, travel duration to distributor’s house in rainy season, how to get repellent, distributor job, transport type owned by distributor, distributor age, knowing distributor, used farm land size and used rice field size. Random effects at both the village and the distributor level were included in the models. The associations between the response variables and the determinants were investigated in a univariate way. Since the contact is a proportion, a binomial error distribution was used. Consumption was modeled using a Gaussian distribution, and given the dependency of the consumption on the availability of the repellent, the contact was included as a covariate in all the models. Operational cost comprising transportation, training, distributors and supervisors was calculated. The analyses for potential determinants of contact were done separately for 2012 and 2013. All analyses were performed in the program R v.3.1.1 [35], using the package nlme [36] and lme4 [37].

### Ethical considerations

This study is a part of the MalaResT project which was approved by the Institutional Review Board of the Institute of Tropical Medicine (Approval IRB/AB/ac/154), the Ethics Committee of University Hospital Antwerp (Approval B300201112714), Belgium and the National Ethics Committee for Health Research of Ministry of Health (Approval 265 NECHR), Cambodia. Household representatives consented verbally to use the repellents and they were routinely informed by distributors about the objectives of repellent distribution and data collection in their local language. In addition they were asked to provide a finger print on the HDS at every bottle exchange contact for proof of contacting the distributor. Every eligible person had his or her own rights to refuse, stop or restart using the repellent anytime without any discussion. Before the start of an interview each participant was informed verbally, either in Khmer for the distributors or in the local languages for the users (mostly via a translator), about the objectives of the surveys, confidentiality of information, anonymity in the database and their rights to deny to participate. Those who agreed to participate in the surveys were asked to stamp a finger print in a top box of the questionnaires.

## Results

### Characteristics of study population

#### Household survey

**Fig. 2:**
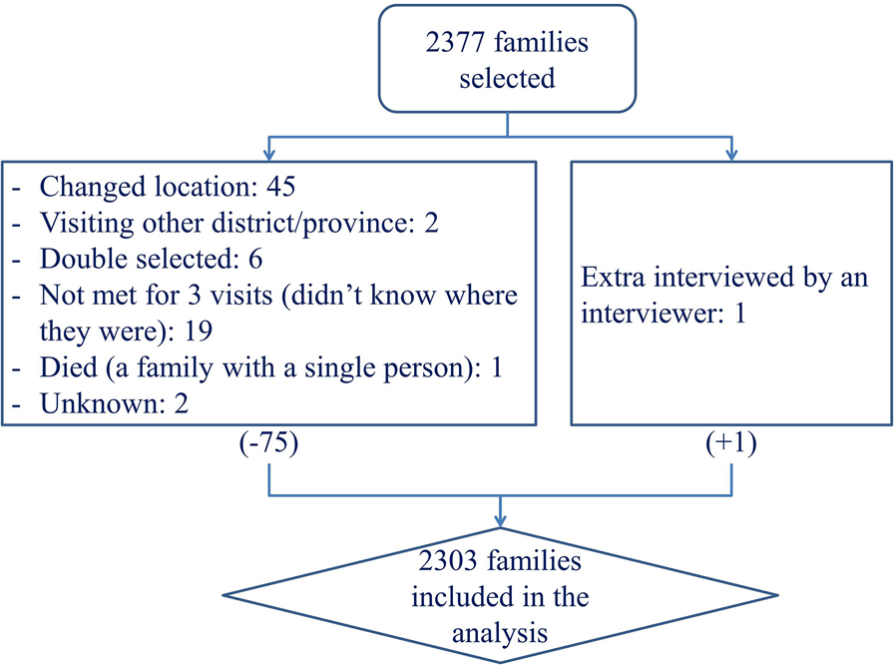
Selection of households and non-responses in the household survey

A total of 2303 households (out of 2377 households selected) were surveyed and included in the analysis. Reasons for not participating are provided in (Fig. 2). Jarai, Tompuon and Kreung were the major ethnicities, each representing about 25 % of the study population, while the remaining households belonged to Kavet, Prov, Cham, Kachok, Lon, Lao and Khmer ethnicities. The majority of households had lived in the area for more than 7 months (99.5 %) and more than half of those consisted of five or more household members including children under five (Median = 5, Q1 = 3, Q3 = 6). Most households owned a wooden (78.7 %) and/or stilted (90.9 %) house, covered with an iron sheet roof (83.2 %). Half of the houses were occupied by two or more nuclear households (Median = 2, Q1 = 1, Q3 = 2). The main occupation of the household heads was farming (92.1 %). Other characteristics are given in Additional file 4.

#### Distributor survey

In 2013, all 135 distributors were surveyed and included in the analysis (Table 1). Since distributors were selected from the community, ethnic distribution matched those of the repellent users: Jarai, Tompuon and Kreung were the major ethnicities among the 135 repellent distributors. Ethnicity of the distributors and user-households was matched in 96.3 % of the cases. The average distributor was a male of about 27 years old, head of the household, and living in the village for over a year. 54.8 % of the distributors had additional roles in the communities such as VMWs (44), VHSGs (13) and others comprising of local authorities and NGO networks (17).

**Table 1:** Distributors’ characteristics obtained from Distributor Survey

Study distributors, N = 135	n	%
Ethnicity		
Jarai	33	24.4
Tompuon	34	25.2
Kreung	35	25.9
Others	33	24.4
Ethnicity user and distributor matched		
Matched	130	96.3
Unmatched	5	3.7
Age		
Median = 27, Q1 = 23.5, Q3 = 38, Min = 16, Max = 70		
Sex		
Male	118	87.4
Female	17	12.6
Status in household		
Family head	94	69.6
Child of family head	28	20.7
Spouse of family head	13	9.6
Main job		
Farmer	124	91.9
Others	11	8.1
Living duration in village (year)		
Median = 23, Q1 = 17, Q3 = 29.5, Min = 1, Max = 70		
Distributor other roles		
Yes	74	54.8
No	61	45.2
Other roles: (N = 74) (Some distributors might have more than one additional role)		
VMW^a^	44	59.5
VHSG^b^	13	17.6
Others (local authorities & NGO networks)	17	23.0

^a^Village Malaria Worker

^b^Village Health Support Group

#### Repellent distribution

##### Accessibility of repellents to households

Almost all household representatives (99.3 %) knew their repellent distributors and mentioned being able (92.5 %) to reach their distributor’s house by motorbike in the rainy season, whereas 4.9 % said they could reach the distributor by boat. 86.1 and 42 % of those who said they could reach the distributor by motorbike and by boat respectively mentioned it took less than 30 min to reach there (Table 2). Of all household representatives, 57.1 % reported that their distributors always visited their houses to distribute repellent, 17.0 % said the reverse and 23.4 % mentioned they went either way.

**Table 2:** Information regarding getting and distributing repellent obtained from Household and Distributor Surveys

Information regarding users, N = 2303	n	%
Knowing repellents distributors: Yes	2287	99.3
Possibility of reaching distributors’ houses in rainy season by motorbike	2287	
Possible	2116	92.5
Travel duration (among “Possible”):	2116	
<30 min	1821	86.1
≥30 min	295	13.9
Possibility of reaching distributors’ houses in rainy season by boat	2287	
Possible	112	4.9
Travel duration (among “Possible”):	112	
<30 min	47	42.0
≥30 min	65	58.0
How to get repellent from distributor		
Distributor always went to my house	1316	57.1
I always went to distributor’s house	392	17.0
Distributor went to my house or I went to his/her house	538	23.4
Others	57	2.5

Information regarding distributors, N = 135	n	%
Number of households responsible: Median = 39, Q1 = 33, Q3 = 45.5		
Distributors having households staying at farm	68	50.4
% families staying at farm: Median = 25.5 %, Q1 = 10 %, Q3 = 68.6 %		
Are the families at farm clustered?	125	
Clustered	12	9.6
Not clustered	84	67.2
Some clustered, some not	29	23.2
Transportations owned by distributor: (A distributor might own more than one item)		
Motorbike	107	79.3
Boat	7	5.2
Bicycle	15	11.1
None	25	18.5
Distributors had no transportation and had households staying at farms	25	
Yes	23	92.0
Those having no transportation, how to distribute repellents to far households	23	
Borrowing transport	14	60.8
Other	9	39.2
Possibility of visiting houses at farm in rainy season: (among those having households staying at farm), N = 125		
Possible	83	66.4
Not possible	12	9.6
Some possible, some not	28	22.4
Don’t know	2	1.6
Duration to the furthest reachable houses: (among “Possible” and “Some possible, some not”), N = 111		
<30 min	7	6.3
30–60 min	44	39.6
>60 min	58	52.3
Duration to the most difficult reachable houses: (among “Possible” and “Some possible, some not”), N = 111		
≥30 min	110	99.1
How to distribute repellent		
I always went to users’ houses	86	63.7
Users always went to my house	1	0.7
Users went to my house or I go to their houses	47	34.8
I distributed to people in the village only	1	0.7

##### Accessibility of households to distributors

In 2013, the median number of households reported per distributor was 39 (Q1 = 33, Q3 = 46) and of those households the median percentage reported staying at the farm per distributor was 25.5 % (Q1 = 10 %, Q3 = 69.4 %). The most common transportation means owned by the distributors was a motorbike (79.3 %), while boat and bicycle were owned by 5.2 and 11.1 % of the distributors, respectively. Among the distributors having no personal transportation (18.5 %), 92 % had user-households staying at farms and 60.8 % of them said they borrowed transportation to bring repellent to the farms. Among all distributors having user-households staying at farms (50.4 %), 66.4 % said they could reach them in the rainy season and about half said they needed more than an hour to reach the farthest farm (Table 2). Most of distributors reported to bring the repellents to users’ houses (63.7 %) while almost all the others (34.8 %) said they did both ways: brought repellents to user’s houses and waited for them at a village central point.

##### Contact rate

Only complete or correct HDSs were taken into account for the analysis (88 % of 46,493 HDSs in 2012 and 93 % of 83,647 HDSs in 2013)

In 2012, the contact rate varied from 0 to 62.5 % per household per year compared to 0 to 136.8 % in 2013. A percentage above 100 % is explained by households exchanging bottles more than once per 2 weeks. In 2012, half of the households had a contact rate of at least 20 % (median = 20 %, Q1 = 12.5 %, Q3 = 33.3 %, range 0–62.5 %) while this increased to more than fourfold in 2013 (median = 88.9 %, Q1 = 73.7 %, Q3 = 94.7 %, range 0–136.8 %) (*p* < 0.001).

##### Determinants for contact

In 2012 district and commune (Additional file 5) and knowing the distributor were significantly associated with contact rate. In 2013 only knowing the distributor remained a significant determinant (Additional file 6). Transport facilities of the distributors did not affect the access to repellents.

#### Repellent consumption

##### Consumption rates

In 2012, the consumption varied from 0 to 83.9 % of the total amount of repellent expected to be consumed by a household per 2 weeks. In 2013, this consumption rate ranged from 0 to 247.4 %. Some people consumed more repellent than expected resulting in a consumption rate over 100 %. In 2012, half of the study households consumed at least 20 % of the expected repellent amount (median = 20.0 %, Q1 = 8.6 %, Q3 = 32.8 %, range 0–90 %) while the consumption rate by household increased nearly threefold in 2013 (median = 57.89 %, Q1 = 37.97 %, Q3 = 79.20 %, range 0–247.4 %) (*p* < 0.001).

**Fig. 3:**
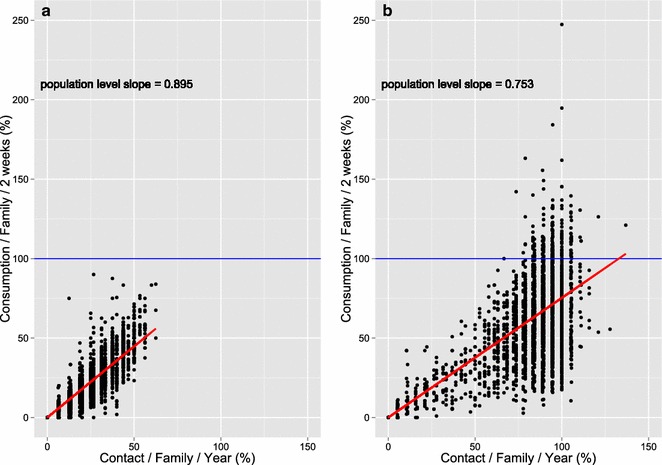
Correlation between contact and consumption in 2012 (**a**) and 2013 (**b**). *Blue lines* indicate expected consumption per household per 2 weeks. An average of 9.0 and 7.5 % increase in consumption for every 10 % increase in contact rates in 2012 and 2013 respectively were observed

Figure 3 shows the correlation between contact and consumption in 2012 and 2013. The fit of a mixed-effects linear regression model is shown, indicating an average of respectively 9.0 and 7.5 % increase in consumption for every 10 % increase in contact rates in 2012 and 2013.

##### Determinants for consumption

**Fig. 4:**
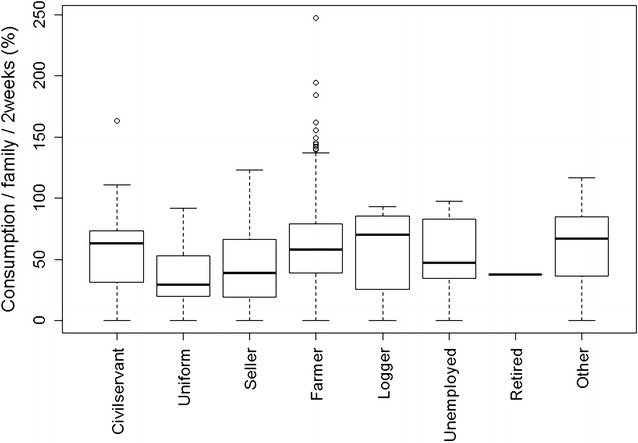
Effect of the occupation of the household head on consumption level in 2013. Households led by farmers significantly consumed more repellent than those led by sellers

Besides the contact rates mentioned above, only two out of 10 determinant variables explored in univariate analysis were significantly associated with consumption in 2013, while none of them were associated with consumption in 2012 (Additional file 7). In 2013, district was significantly associated with consumption (*p* = 0.0454). On average the consumption was highest in Taveng and Kom Mom districts compared to other districts (Additional file 8). In the same year, consumption was significantly influenced by “user’s household head’s occupation” (*p* = 0.0166). The households led by farmers are more likely to consume more repellent than those led by sellers (Fig. 4). Only eight interviewees mentioned their household was led by loggers. Determinants related to the distributors were not associated with consumption.

The annual operational cost per capita for repellent distribution was about 31 times more expensive than that for LLINs (USD 4.33 versus USD 0.14) (Table 3).

**Table 3:** Operational costs for repellents and LLINs distribution in the study setting

Cost item	Annual cost per capita	
	Repellents distribution	LLINs distribution
Transportation	USD 0.79	USD 0.09
Training for distributors	USD 0.14	Not applicable
Incentive/per diem for distributors and supervisors	USD 3.40	USD 0.05
*Total*	*USD 4.33*	*USD 0.14*

Annual cost per person for repellents distribution compared with LLINs having a lifetime of 3 years

## Discussion

The current study reports on an intensive distribution and follow-up system of repellents, built on the existing health system of community health worker in Cambodia. It demonstrated that, given these well-supervised trial conditions, mass distribution was achieved. A fourfold increase in the contact rates between the repellent distributor and the households in the community and about a threefold increase in the consumption rates was documented in the second year. This increase was associated with more intensive efforts to improve the distribution and use of repellent in the second year of study, such as the recruitment of additional distributors and supervisors, and the increase of logistic and financial supports to distributors. Moreover, more attractive health education campaigns (consisting of a movie show in the communities) followed by door-to-door health education by the supervisors in 2013 were carried out.

Although contact and consumption were correlated, the variance around the correlation increased with higher contact rates (Fig. 3). This illustrates that increasing ownership of vector control tools is easier than increasing its actual use, which was also shown in the context of bed net distribution [38]. As such, both the intensive distribution system and the health promotion should run parallel to ensure a performant system in terms of ownership and use [39–41].

Earlier studies demonstrated that socio-economic factors influence ownership and use of malaria vector control tools. However these control measures were not provided free of charge [24]. In contrast, in the present study, no relation was observed between SES and the contact and consumption rates, suggesting that equity of ownership was obtained using a free distribution strategy, characterized by a 2-weekly repellent exchange schedule, and implemented by re-enforcing the existing VMW and VHSG systems with additional human and financial resources.

Contact rates were observed to be higher in both study years in households that reported to know the distributor as their relative, friend or neighbour. This is an expected but important element of intensive distribution schemes that might be further exploited in the implementation of such systems. A similar result was obtained in a study on condom use through a condom coupon redemption programme in Kampala, Uganda. The most popular condom distributors in both intervention and control arms redeemed also the most condom coupons [42]. It should be noted that the contact and consumption rates in the present study were probably underestimated as 12 and 7 % of the Household Datasheets for 2012 and 2013 respectively were incomplete and not considered for analysis. Moreover during parallel observational studies in the villages it was occasionally reported that people received repellents from the distributors without being recorded in HDS (Gryseels et al, personal communication).

Significant variation in contact per district was observed in 2012, but not in 2013. In 2012, in two districts (Lum Phat and Oyadao) distribution was less performant as compared to the other districts (Additional file 5). The study protocol expected each distributor to spend at least 2 days per 2 weeks to distribute the repellents to all households before meeting at the health centers to replenish stock. In 2012, they did this on a voluntary basis, receiving only a small per-diem and gasoline for travelling in the village. Working on a voluntary basis caused large variations in the amount of effort of the distributors in arranging repellent exchanges with villagers, which later resulted in large variation from one commune to another and from one district to another. This was confirmed by the absence of association between district or commune and contact rates in 2013 after replacing inactive distributors who were busy with their own private businesses and improving distributors’ supports and supervision system.

District was also significantly associated with consumption in 2013. Three districts (Kon Mom, O Chum and Taveng) showed higher consumption rates than the others (Additional file 8). Possibly the study villages in these districts were more disturbed by insect nuisance than the others (Gryseels et al. personal communication). In 2013, consumption was significantly associated with household head occupation. The households led by farmers were more likely to consume more repellent than those led by sellers (Fig. 4). In this context, the sellers usually stay in the village for business while the farmers work or stay near or in the forest where they are probably more exposed to insect nuisance leading them to consume more repellents than others [43]. Likely loggers would be the most exposed to insects as they spend most of the time in the forest. However, their number was too limited (8) to find a significant association. The SES was not significantly related to the consumption rates, which suggests that there might be other factors (which were not measured in the study) that are more related to use. Indeed, a mixed methods study on repellent use performed in parallel to the present study concluded that repellent acceptability was high but that repellent use depended on other variables such as location of use (the deep forest, the forest farm), seasonal and economic activities, and level of insect nuisance (Gryseels et al. personal communication). Personal preference factors, such as perceived smell of the repellent and fear of side-effects were, especially among children and women, the main causes for not using repellents (Gryseels et al. personal communication). This suggests that, in the context of malaria elimination, making intervention tools available alone will not be sufficient to ensure use. Moreover, consumption of repellent does not necessarily mean correct use, as users reported several alternative uses (spray on the insects, around the body, on clothes, on nets) instead of applying the product on the exposed skin surfaces (Gryseels et al. personal communication).

The annual cost per capita for repeated repellent distribution and follow up is much more expensive than the one for LLINs. However given that repellent distribution is expected to last only during a period of intensified control efforts to eliminate malaria in a given area, these extra costs may be fully justified.

In the context of Cambodia, where a strong community-based malaria control system (VMW) [31] is an effective means to make malaria diagnosis and treatment accessible to people in remote areas [44], adding other health services to the system did not degrade its quality [28], because VMW’s basic scope of work is very small [29]. Therefore, adding more tasks, incentive and adequate programmatic supports to the VMWs and VHSGs (such as repellent distribution in the current study) can be a way of motivating community health worker s to work efficiently in a comprehensive malaria control programme, or an integrated control programme comprising also of other diseases. Indeed, intensive distribution systems could also be useful for other personal protection tools. However increasing responsibilities of community health workers or VMWs will have cost implications and cost-effectiveness of this approach should be evaluated in the context of an elimination strategy. Moreover, additional ways of implementing such a repellent distribution should be explored, for example through commercial and social channels such as schools, agricultural and religious networks to make the product accessible to rural users.

## Conclusions

A 2-weekly repellent distribution system is an intensive scheme that ensures access to the tool for the entire target population. In the present study it was shown that such an intensive distribution scheme is feasible when built on the existing community health worker system and providing additional incentives, logistical and programmatic support. Health education campaigns were conducted in parallel. As such, intensive distribution systems, which are needed in the final run for malaria elimination, can be deployed in low resource countries, taking into account the effectiveness of the vector control measure provided and time until elimination is expected to be reached. Moreover, such systems can at the same time be used for distribution of other disease intervention tools. The present study has shown that, in similar contexts, the distributors should be selected from a well-functioning existing community-based health network, such as the VMWs and VHSGs in this study and complemented by volunteers selected among local authorities, community NGO networks and ultimately local people. Alternative or supplementary distribution channels should be considered. In the present study the SES of users had no influence on access to and consumption of repellents which pleads for free distribution of disease control tools by national programmes to achieve equity which may not necessarily be achieved using commercial channels.

## Additional files

10.1186/s12936-015-0960-4 Form for distribution of repellent bottles and recovery of empty bottles or household data sheet. This sheet was used to collect information from household representative during two-weekly bottle exchange. Each sheet is for each bottle exchange and for a household. Each household had a unique identification code (family code) which was used during the entire project.
10.1186/s12936-015-0960-4 Questionnaire for repellent users for socio-economic status and repellents distribution survey: A Khmer version of this questionnaire was used during the household survey to collect information on socio-economic status of selected households and how they got repellents from distributors. The same family code used in household data sheet was use for this survey.
10.1186/s12936-015-0960-4 Questionnaire for distributors for socio-economic status and repellents distribution survey. A Khmer version of this questionnaire was used during the distributor survey to collect information on socio-economic status of all distributors and how they distribute repellents.
10.1186/s12936-015-0960-4 Characteristics of households included in the Household Survey. The table summarizes potential variables to be included in the principal component analysis for socio-economic status which is possible to influence on distributor-household contact and repellent consumption.
10.1186/s12936-015-0960-4 Differences in contact level between districts (left) and communes (right) in 2012. The figures detail districts and their corresponding communes which are associated with distributor-household contact in 2012. The distribution of repellents in district Lum Phat and Oyadao was less performant as compared to the others.
10.1186/s12936-015-0960-4 Univariate analysis of potential determinants for contact. The table shows all results of univariate analysis looking for relationship between each of ten potential determinants and distributor-household contact in 2012 and 2013.
10.1186/s12936-015-0960-4 Univariate analysis of potential determinants for consumption. The table shows all results of univariate analysis looking for relationship between each of ten potential determinants and average two-weekly repellent consumption in 2012 and 2013.
10.1186/s12936-015-0960-4 Differences in consumption levels between districts in 2013. The figure details districts that are associated with repellent consumption in 2013. The average repellent consumption was highest in Taveng and Kom Mom districts compared to others.
