# Supplementary material for: Assuring access to topical mosquito repellents within an intensive distribution scheme: a case study in a remote province of Cambodia
Source: Malar J. 2015 Nov 24;14:468. doi: 10.1186/s12936-015-0960-4 (PMC4657324; doi:10.1186/s12936-015-0960-4)
Supplement: Supplementary file 1 — 10.1186/s12936-015-0960-4 Form for distribution of repellent bottles and recovery of empty bottles or household data sheet. This sheet was used to collect information from household representative during two-weekly bottle exchange. Each sheet is for each bottle exchange and for a household. Each household had a unique identification code (family code) which was used during the entire project. [file 12936_2015_960_MOESM1_ESM.pdf]

Additional file 1

Distribution of repellent bottles and recovery of empty bottles

Village: ____________ Commune:____________ Family code: I___I___I___I___I/I___I___I___I___I

No.	Family member code	Recovery empty bottles				ID new bottle	Date dd/mm/yy
		ID bottle	Amount still in the bottle (ml)	Reported side effects	If repellent is not used, ask why?		
1	I__I__I	I__I__I I__I__I__I__I__I__I				I__I__I I__I__I__I__I__I__I	I__I__I/ I__I__I/ I__I__I
2	I__I__I	I__I__I I__I__I__I__I__I__I				I__I__I I__I__I__I__I__I__I	I__I__I/ I__I__I/ I__I__I
3	I__I__I	I__I__I I__I__I__I__I__I__I				I__I__I I__I__I__I__I__I__I	I__I__I/ I__I__I/ I__I__I
4	I__I__I	I__I__I I__I__I__I__I__I__I				I__I__I I__I__I__I__I__I__I	I__I__I/ I__I__I/ I__I__I
5	I__I__I	I__I__I I__I__I__I__I__I__I				I__I__I I__I__I__I__I__I__I	I__I__I/ I__I__I/ I__I__I
6	I__I__I	I__I__I I__I__I__I__I__I__I				I__I__I I__I__I__I__I__I__I	I__I__I/ I__I__I/ I__I__I
7	I__I__I	I__I__I I__I__I__I__I__I__I				I__I__I I__I__I__I__I__I__I	I__I__I/ I__I__I/ I__I__I
8	I__I__I	I__I__I I__I__I__I__I__I__I				I__I__I I__I__I__I__I__I__I	I__I__I/ I__I__I/ I__I__I
9	I__I__I	I__I__I I__I__I__I__I__I__I				I__I__I I__I__I__I__I__I__I	I__I__I/ I__I__I/ I__I__I
10	I__I__I	I__I__I I__I__I__I__I__I__I				I__I__I I__I__I__I__I__I__I	I__I__I/ I__I__I/ I__I__I
Total							

Signature/finger print of family representative

Signature/finger print of distributor
