# Supplementary material for: Assuring access to topical mosquito repellents within an intensive distribution scheme: a case study in a remote province of Cambodia
Source: Malar J. 2015 Nov 24;14:468. doi: 10.1186/s12936-015-0960-4 (PMC4657324; doi:10.1186/s12936-015-0960-4)
Supplement: Supplementary file 2 — 10.1186/s12936-015-0960-4 Questionnaire for repellent users for socio-economic status and repellents distribution survey: A Khmer version of this questionnaire was used during the household survey to collect information on socio-economic status of selected households and how they got repellents from distributors. The same family code used in household data sheet was use for this survey. [file 12936_2015_960_MOESM2_ESM.pdf]

Additional file 2

Socio-economic status and repellents distribution survey
Questionnaire for repellent users

Family code: |__|__|__|__|/|__|__|__|__|

Informed consent:

Hello, my name is ______________________ from _________________health center, I'm working for MalaResT project (repellent project). I would like to disturb you for about 20 minutes asking some questions regarding your or your family head job, your house, your assets and how you get repellents. You have full right to deny answering any questions or stop being interviewed at any times by just telling us, you won't have any problem. Your name and information you provide us are kept secret, only your family code number will be used for data analysis and will not be displayed anywhere else. Your information is very crucial for us and the National Malaria Center in Cambodia to look for better strategies to improve repellents coverage in your community.

Should I start interviewing you now?
☐ Yes. Start the interview
☐ No. Stop interview

Name of interviewer: ________________________________
Date of interview: _______/________/2013

General information:

1. Address of household:
    - District:_________________
    - Commune:______________
    - Village:_________________
2. Place of the interview:
   ☐ 1) Village ☐ 2) Farm ☐ 3) Field
3. Name and surname of interviewee (adult): _______________________
4. Age of interviewee: |__|__|years ☐ 99) Don't know
5. Gender of interviewee:
   ☐ 1) Male ☐ 2) Female
6. Ethnicity:
   ☐ 1) Khmer ☐ 2) Kroeng ☐ 3) Tumpoun ☐ 4) Charay ☐ 5) Prouv
   ☐ 6) Cham ☐ 7) Kachak ☐ 8) Lun ☐ 9) Lao ☐ 10) Kavet
   ☐ 11) Other, specify: _________________
7. Status in the family:
   ☐ 1) Head ☐ 2) Spouse of family head ☐ 3) Child of family head
   ☐ 4) Other, specify: ___________

8. How long your family lives in this village?
   ☐ 1) About 7 months ☐ 2) Less than 7 months
   ☐ 3) More than 7 months ☐ 99) Don't know
9. How many people are there in your family? |__|__| people
10. Of which how many are children under 5? |__|__| children under 5
11. House many houses does your family have (including those in farms or rice fields)?
    |__| houses

(Put "0" if no house or stay with friend or relative. Staying with parents is considered as having a house).

[*If "0"to question "12", otherwise skip to question "13"*]

12. If "0", where are you staying?
    ☐ 1) With friend or relative ☐ 2) In a rent house
    ☐ 3) Other, specify: ____
    [***Skip to question "22"]***

House characteristic:

Definition of a family: *a group of people with the same permanent address that eat together by sharing rice-pot or spending money together for meal.*

13. Status of house observed? ☐ 1) Yes ☐ 2) No
14. Type of main part of the house where the family spends most of their times or they consider as their permanent house:
    ☐ 1) On pillar ☐ 2) On ground ☐ 3) Other, specify: ____________________
15. Type of wall: (multiple answers is possible)
    ☐ 1) Brick ☐ 2) Wood ☐ 3) Tree bark ☐ 4) Bamboo
    ☐ 5) Thatch ☐ 6) Plastic ☐ 7) Iron sheet ☐ 8) Other, specify: _____________
16. Type of roof: (multiple answers is possible)
    ☐ 1) Tile ☐ 2) Iron sheet ☐ 3) Thatch ☐ 4) Other, specify: _____________
17. Completeness of wall (excluding open window):
    ☐ 1) Complete ☐ 2) Incomplete
18. Completeness of roof:
    ☐ 1) Complete ☐ 2) Incomplete
19. General condition of the house:
    ☐ 1) Old and about to collapse ☐ 2) Medium can be lived in ☐ 3) Good condition
20. House size?
    ☐ 1) 20 m^2 or less ☐ 2) 21-50 m^2 ☐ 3) 51 m^2 or more
21. How many families live in this house? |__|__| families (put "01" if only interviewee family living in this house)

Job:

22. What is the main job of your family head?
    ☐ 1) Civil servant ☐ 2) Uniform ☐ 3) Seller
    ☐ 4) Farmer ☐ 5) Logger ☐ 6) Unemployed
    ☐ 7) Retired ☐ 8) Other, specify: ____________________

Assets:

23. What kinds of transportation means does your family own? (multiple answers is possible)
    ☐ 1) Car ☐ 2) Truck ☐ 3) Small truck
    ☐ 4) Boat ☐ 5) Motorbike ☐ 6) Bicycle ☐ 99) None
24. What kinds of agricultural means does your family own? (multiple answers is possible)
    ☐ 1) Tractor ☐ 2) Plowing machine ☐ 3) Grass cutting machine
    ☐ 4) Grass spraying machine ☐ 5) Rice/bean mill ☐ 99) None
25. What kinds of animal does your family own? (multiple answers is possible)
    ☐ 1) Cow: number: |__|__|
    ☐ 2) Buffalo: number: |__|__|
    ☐ 3) Pig: number: |__|__|
    ☐ 4) Chicken: number: |__|__|
26. What kinds of entertainment material does your family own? (multiple answers is possible)
    ☐ 1) Television ☐ 2) DVD player ☐ 3) DTV antenna
    ☐ 4) Laptop DVD player ☐ 5) Radio ☐ 6) Mobile phone ☐ 99) None
27. What kinds of light or power sources does your family use? (multiple answers is possible)
    ☐ 1) Paid public electricity ☐ 2) Free electricity from parents/relatives/neighbors…
    ☐ 3) Own generator ☐ 4) Own solar panel ☐ 5) Own battery
    ☐ 6) Own petrol lamp ☐ 7) Own battery lamp
    ☐ 8) Other, specify: ____________________
28. How many hectares of **farm** land is your family cultivating on? (including rent and non-rent farm land, e.g. land from friend or relative)
    |__|__|__| hectares,|__|__|__| Rais (1 Rai=1600m^2) ☐ 99) Don't know
29. How many hectares of **rice field** is your family cultivating on? (including rent and non-rent rice field, e.g. rice field from friends or relatives)
    |__|__|__| hectares,|__|__|__| Rais (1 Rai=1600m^2) ☐ 99) Don't know
30. How many hectares of productive cashew nut farm does your family own?
    |__|__|__| hectares,|__|__|__| Rais (1 Rai=1600m^2) ☐ 99) Don't know
31. What is the most appropriate transportation means to take from your main house (refer to the house in question "14") to the furthest farm/rice field in the rainy season? **[Skip this question to question "33" if "28 + 29" are "0"]**
    ☐ 1) Boat ☐ 2) Motorbike ☐ 3) Bicycle ☐ 4) On foot
    ☐ 5) Other, specify: ___________ ☐ 6) Motorbike & boat ☐99) Don't know
32. How long it takes from your main house (refer to the house in question "14") to the furthest farm/rice field in the rainy season by using transportation means in question "31"?
    ☐ 1) Less than 30 minutes ☐ 2) 30-60 minutes ☐ 3) More than 60 minutes
    ☐ 99) Don't know

Distance and road condition to distributor house and ways of bottle exchanges:

33. Do you know who currently distributes repellents to your family?
    ☐ 1) Yes ☐ 2) No ***[skip to question "35"]***
34. If "Yes", what is his/her name? __________________________
35. Where are you currently staying?
    ☐ 1) Farm house ☐ 2) Field house ☐ 3) Village house

36. From this house, is it possible to go to the house where the distributor keeps the repellents in the rainy season by motorbike?
    ☐ 1) Yes
    ☐ 2) No ***[skip to question "38"]***
    ☐ 99) Don't know ***[skip to question "38"]***
37. If "Yes", how long it takes from the house you are staying now to the distributor's house where the repellents are stored in rainy season by motorbike?
    ☐ 1) Less than 30 minutes ☐ 2) 30-60 minutes ☐ 3) More than 60 minutes
    ☐ 99) Don't know
38. Is it possible to go by boat?
    ☐ 1) Yes ☐ 2) No ***[skip to question "40"]***
    ☐ 99) Don't know ***[skip to question "40"]***
39. If "Yes", how long it takes from the house you are staying now to the distributor's house where the repellents are stored in rainy season by boat?
    ☐ 1) Less than 30 minutes ☐ 2) 30-60 minutes ☐ 3) More than 60 minutes
    ☐ 99) Don't know
40. Usually, how do you get the repellents at the place where you are staying now?
    ☐ 1) Distributor brings repellents to my house.
    ☐ 2) I go to take repellents from distributor's house.
    ☐ 3) Sometime distributor comes to my house, sometime I go to distributor's house.
    ☐ 4) I never got repellents or I haven't got repellent for a long time.
    ☐ 5) Others, specify: ____
    ☐ 99) Don't know

Many thanks for your participation!!
