# Supplementary material for: Assuring access to topical mosquito repellents within an intensive distribution scheme: a case study in a remote province of Cambodia
Source: Malar J. 2015 Nov 24;14:468. doi: 10.1186/s12936-015-0960-4 (PMC4657324; doi:10.1186/s12936-015-0960-4)
Supplement: Supplementary file 3 — 10.1186/s12936-015-0960-4 Questionnaire for distributors for socio-economic status and repellents distribution survey. A Khmer version of this questionnaire was used during the distributor survey to collect information on socio-economic status of all distributors and how they distribute repellents. [file 12936_2015_960_MOESM3_ESM.pdf]

Additional file 3

Socio-economic status and repellents distribution survey
Questionnaire for distributors

Distributor code: |__|__|__|__|/|__|__|

Informed consent:

Hello, my name is ____________________ from MalaResT project (repellent project). I would like to disturb you for about 10 minutes asking some questions regarding your job, your role(s) in the community and the ways you are distribute repellents. You have full right to deny answering any questions or stop being interviewed at any times by just telling us, you won't have any problem. Your name and information you provide us are kept secret, only your code number will be used for data analysis and will not be displayed anywhere else. Your information is very crucial for us and the National Malaria Center in Cambodia to look for better strategies to improve the coverage of repellents in your community.

Should I start interviewing you now?

☐ Yes. Ask for a finger print and start the interview

☐ No. Stop interview

Name of interviewer: ____________________________
Date of interview: _______/________/2013

General information:

1. Address of distributor:
   - District:________________
   - Commune:_____________
   - Village:________________
2. Name and surname of interviewee: ______________________
3. Age of interviewee: |__|__|years ☐ 99) Don't know
4. Gender of interviewee:
   ☐ 1) Male ☐ 2) Female
5. Ethnicity of interviewee:
   ☐ 1) Khmer ☐ 2) Kroeng ☐ 3) Tumpoun ☐ 4) Charay ☐ 5) Prouv
   ☐ 6) Cham ☐ 7) Kachak ☐ 8) Lun ☐ 9) Lao ☐ 10) Kavet
   ☐ 11) Other, specify: ________________
6. What is the main ethnicity of your villagers?
   ☐ 1) Khmer ☐ 2) Kroeng ☐ 3) Tumpoun ☐ 4) Charay ☐ 5) Prouv
   ☐ 6) Cham ☐ 7) Kachak ☐ 8) Lun ☐ 9) Lao ☐ 10) Kavet
   ☐ 11) Other, specify: ________________

7. Status in the family:
   ☐ 1) Family head ☐ 2) Spouse of head ☐ 3) Child of family head
   ☐ 4) Other, specify: _______________ _____
8. How long have you been living in this village? |__|__| years
9. Besides a repellent distributor do you have any other role(s) in your community?
   ☐ 1) Yes ☐ 2) No ***[skip to question "11"]***
10. If "Yes", what is that or those role(s)?
    1) ______________________________________
    2) ______________________________________
    3) ______________________________________
11. What is your main job?
    ☐ 1) Civil servant ☐ 2) Seller ☐ 3) Farmer ☐ 4) Logger ☐ 5) Unemployed
    ☐ 6) Retired ☐ 7) Other, specify: _______________________
12. How many families are you responsible for distributing repellents? |__|__| families
13. How many of your families are staying at the farms? |__|__| families ***[if "0" skip to question "15"]***
14. Are the families at the farms clustered in a hamlet?
    ☐ 1) Yes ☐ 2) No ☐ 3) Some clustered, some scattered ☐ 99) Don't know
15. How many of your families are staying in the village? |__|__| families
16. Where are you currently staying?
    ☐ 1) In the village ☐ 2) In the farm/field
17. What transportation means do you have? (multiple answers)
    ☐ 1) Motorbike ☐ 2) Boat ☐ 3) Bicycle
    ☐ 4) Others, specify: ______________ ☐ 99) None ***[to question "18", otherwise skip to "19"}***
18. If **"None"** how do you do when you need transportation means to distribute repellents?
    ☐ 1) Borrow the others ☐ 2) Ask other distributor(s) to do instead
    ☐ 3) Just don't distribute ***[skip to question "22"]***
    ☐ 4) Have no far family needing transportation means ***[skip to question "22"]***
    ☐ 5) Other, specify: ______________________________
19. Is it possible to visit the houses at the farms in rainy season? ***[skip this question to "22"if question "13" ="0"]***
    ☐ 1) Yes ☐ 2) No ***[skip to question "22"]*** ☐ 3) Some possible, some not
    ☐ 99) Don't know ***[skip to question "22"]***
20. If **"Yes"** or **"Some possible, some not"** , how long it takes to the furthest houses?
    ☐ 1) Less than 30 minutes ☐ 2) 30-60 minutes
    ☐ 3) More than 60 minutes ☐ 99) Don't know
21. If **"Yes"** or **"Some possible, some not",** how long it takes to the most difficult houses?
    ☐ 1) Less than 30 minutes ☐ 2) 30-60 minutes
    ☐ 3) More than 60 minutes ☐ 99) Don't know
22. How do you distribute the repellents?
    ☐ 1) I bring repellents to villagers' houses.
    ☐ 2) Villagers come to take the repellents from my house.
    ☐ 3) Sometime villagers come to my house, sometime I go to their houses.
    ☐ 4) I only bring to those people who stay in the village.

☐ 5) Others, specify: ____

Many thanks for your participation
