# Supplementary material for: Assuring access to topical mosquito repellents within an intensive distribution scheme: a case study in a remote province of Cambodia
Source: Malar J. 2015 Nov 24;14:468. doi: 10.1186/s12936-015-0960-4 (PMC4657324; doi:10.1186/s12936-015-0960-4)
Supplement: Supplementary file 4 — 10.1186/s12936-015-0960-4 Characteristics of households included in the Household Survey. The table summarizes potential variables to be included in the principal component analysis for socio-economic status which is possible to influence on distributor-household contact and repellent consumption. [file 12936_2015_960_MOESM4_ESM.pdf]

Additional file 4: *Characteristics of families included in the Household Survey*

**Study families, N=2303**			**n**	**%**
Ethnic groups:		- Jarai	585	25.4
		- Tompuon	569	24.7
		- Kreung	565	24.5
		- Others	584	25.4
Living duration in village:		- ≤7 months	9	0.4
		- >7 months	2292	99.5
		- Don't know	2	0.1
Family size:		Median=5, Q1=3, Q3=6		
Number of under 5 children:		Median=1, Q1=0, Q3=1		
Number of houses:		Median=2, Q1=1, Q2=2		
Main house characteristics:*(Among families that had houses, N=2246)*				
	House type:	- On pillar	2042	90.9
		- On ground	204	9.1
	Wall type:	- Wood	1767	78.7
		- Bamboo	141	18.4
	Roof type:	- Iron sheet	1869	83.2
		- Thatch	178	7.9
	Wall complete:	- Yes	1900	84.6
	Roof complete:	- Yes	2156	96.0
	House condition:	- Good	1348	60.0
		- Medium	794	35.4
		- Old & about to collapse	104	4.6
	House size:	- ≤ 20 m^2	669	29.8
		- 21-50 m^2	1149	51.2
		- ≥51 m^2	428	19.1
Number of families per house:		(Among those having houses, N=2243, missing=3) (Median=2, Q1=1, Q3=2)		
Family head occupation:		- Farmer	2121	92.1
Transportation: *(some families might own more than one item, N=2302, missing=1)*		- Motorbike	1738	75.5
		- Bicycle	394	17.1
		- None	458	19.9
Agriculture equipment: *(Some families might own more than one item)*		- Grass cuter	801	34.8
		- Grass sprayer	321	13.9
		- Rice or bean mill	193	8.4
		- None	1326	57.6
Size of used farm land (Ha):		Median=1, Q1=0.5, Q3=2 (N=2292, missing=11)		
Size of used rice field (Ha):		Median=0, Q1=0, Q3=1 (N=2299, missing=4)		
Size of cashew farm (Ha):		Median=0, Q1=0, Q3=1 (N=2299, missing=4)		
Number of cows:		Median=0, Q1=0, Q3=1		
Number of buffaloes:		Median=0, Q1=0, Q3=1		
Number of pigs:		Median=1, Q1=0, Q3=2		
Number of chickens:		Median=2, Q1=0, Q3=5		
Communication material:		- Mobile phone	1587	68.9
Entertainment material: *(Some families might own* more than one item)		- Radio	809	35.1
		- Television	468	20.3
		- DVD player	392	17.0
		- Laptop DVD player	278	12.1
		- DTV antenna	49	2.1
		- None	466	20.2
Lighting material:		- Battery lamp	1389	60.3
Power sources: *(Some families might own more than one item)*		- Battery	731	31.7
		- Generator	370	16.1
