# Supplementary figures and images for: Assuring access to topical mosquito repellents within an intensive distribution scheme: a case study in a remote province of Cambodia
Source: Malar J. 2015 Nov 24;14:468. doi: 10.1186/s12936-015-0960-4 (PMC4657324; doi:10.1186/s12936-015-0960-4)

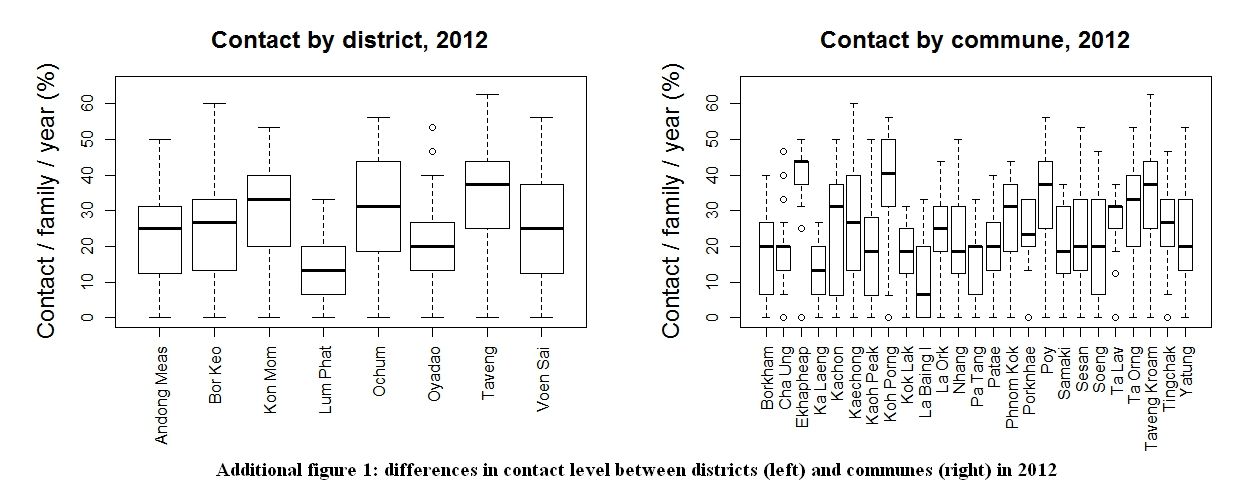

Supplement: Supplementary file 5 — 10.1186/s12936-015-0960-4 Differences in contact level between districts (left) and communes (right) in 2012. The figures detail districts and their corresponding communes which are associated with distributor-household contact in 2012. The distribution of repellents in district Lum Phat and Oyadao was less performant as compared to the others. [file 12936_2015_960_MOESM5_ESM.jpeg]

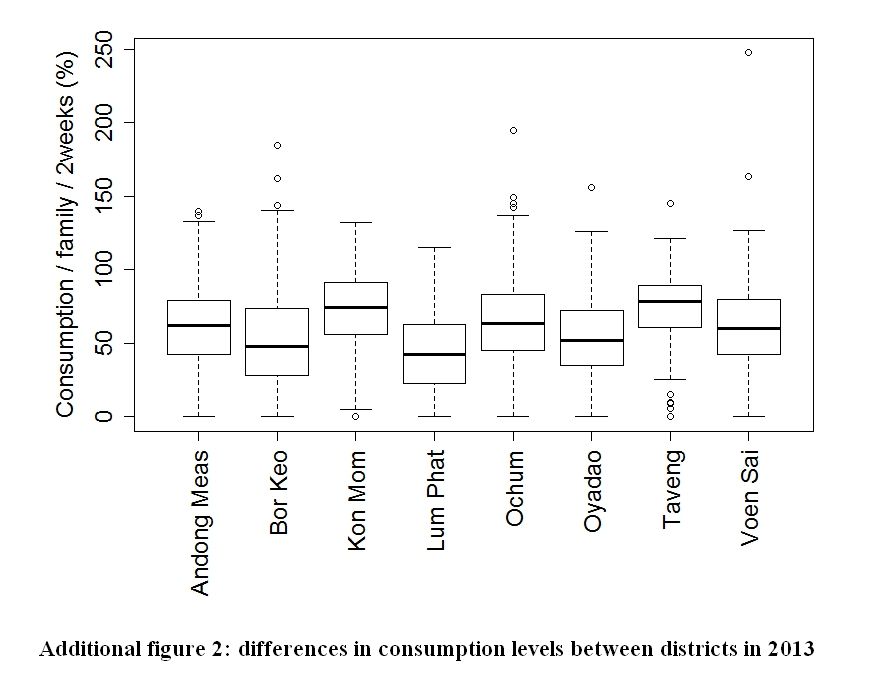

Supplement: Supplementary file 8 — 10.1186/s12936-015-0960-4 Differences in consumption levels between districts in 2013. The figure details districts that are associated with repellent consumption in 2013. The average repellent consumption was highest in Taveng and Kom Mom districts compared to others. [file 12936_2015_960_MOESM8_ESM.jpeg]
