# Supplementary material for: Assuring access to topical mosquito repellents within an intensive distribution scheme: a case study in a remote province of Cambodia
Source: Malar J. 2015 Nov 24;14:468. doi: 10.1186/s12936-015-0960-4 (PMC4657324; doi:10.1186/s12936-015-0960-4)
Supplement: Supplementary file 6 — 10.1186/s12936-015-0960-4 Univariate analysis of potential determinants for contact. The table shows all results of univariate analysis looking for relationship between each of ten potential determinants and distributor-household contact in 2012 and 2013. [file 12936_2015_960_MOESM6_ESM.pdf]

Additional file 5: *Univariate analysis of potential determinants of contact*

**Variables**	**2012**		**2013**	
	Xi²	***P-value***	***Xi²***	***P-value***
Socio-economic status	3.61	0.4613	3.59	0.4645
District	26.42	**0.0004**	4.34	0.7395
Commune	49.32	**0.0017**	26.81	0.3135
User's family head occupation	1.82	0.1775	2.6	0.107
Travel duration to distributor's house in rainy season	3.3588	0.3395	3.8592	0.2771
How to get repellent	0.3307	0.8476	3.0326	0.2195
Distributor job	4.22	0.3765	2.33	0.6745
Transport type owned by distributor	12.14	0.0589	5.38	0.4965
Distributor age	2.21	0.331	1.03	0.598
Knowing distributor	10.595	**0.0011**	11.269	**0.0008**
