# Supplementary material for: Assuring access to topical mosquito repellents within an intensive distribution scheme: a case study in a remote province of Cambodia
Source: Malar J. 2015 Nov 24;14:468. doi: 10.1186/s12936-015-0960-4 (PMC4657324; doi:10.1186/s12936-015-0960-4)
Supplement: Supplementary file 7 — 10.1186/s12936-015-0960-4 Univariate analysis of potential determinants for consumption. The table shows all results of univariate analysis looking for relationship between each of ten potential determinants and average two-weekly repellent consumption in 2012 and 2013. [file 12936_2015_960_MOESM7_ESM.pdf]

Additional file 6: *Univariate analysis of potential determinants of consumption*

**Variable**	**2012**		**2013**	
	**LRT**	***P-value***	**LRT**	***P-value***
Socio-economic status	0	1	0	1
District	0	1	14.34	**0.0454**
Commune	0.02	1	32.35	0.1184
User's family head occupation	2.07	0.9556	17.13	**0.0166**
Used farm land size	0	0.9813	0	0.9542
Used rice field size	0	0.9821	0.03	0.8631
How to get repellent	0	0.9997	0	0.9992
Distributor age	0	0.9999	3.21	0.2005
Transport type owned by distributor	0.71	0.9943	11.85	0.0654
Knowing distributor	0	0.9993	0.48	0.4871
